# Self-Deception in Clinical Nursing Practice: A Concept Analysis

**DOI:** 10.1177/10547738231206610

**Published:** 2023-10-23

**Authors:** Granville Eric Miller, Dave Holmes

**Affiliations:** 1 University of Ottawa, ON, Canada

**Keywords:** concept analysis, clinical practice, nursing, power, self-deception

## Abstract

In this paper, we explore the phenomenon of “self-deception” within the context of nursing, focusing on how nurses employ this coping mechanism when faced with dissonance, distress, and conflicting situations in clinical settings. Our primary objective is to examine the phenomenon of self-deception using Rodgers’ evolutionary method of concept analysis. Focusing on nurses’ experiences in challenging situations, our analysis highlights how self-deception is often employed as a coping strategy. According to our conceptual analysis, self-deception in nursing clinical practice highlights tensions between different paradigms and expectations in healthcare settings. These tensions stem from the power dynamics and subservience that nurses often face, which can hinder their ability to advocate for themselves, their patients, and the nursing profession.

## Introduction

This paper seeks to explore the phenomenon of “self-deception” within the context of nursing, focusing on how nurses employ this coping mechanism when faced with dissonance, distress, and conflicting situations in clinical settings. Through this exploration, our aim is to foster our understanding of self-deception, ultimately contributing to the mitigation of its adverse effects. This, in turn, holds the potential to elevate the quality of practice and patient care. Nurses can leverage this understanding to drive positive transformations, champion nurse advocacy, and optimize health systems.

Concept analysis holds a profound significance within the realm of nursing, serving as a critical tool for advancing understanding, communication, and practice. The complexities inherent in nursing concepts necessitate a clear and coherent framework for their exploration, development, and application. As the landscape of nursing knowledge constantly evolves, concept analysis offers a structured path to navigate the myriad challenges posed by concept development (Knafl & Rodgers, 2000). The process of concept analysis in nursing is not only about unveiling definitions but it is about shaping the foundation of nursing practice, education, and research. As nursing grapples with ethical dilemmas, evolving healthcare dynamics, and interdisciplinary collaboration, the significance of concept analysis becomes even more pronounced. By fostering a solid philosophical foundation and encouraging critical thinking, concept analysis empowers nurses to make informed decisions that uphold ethical standards and contribute to the holistic well-being of patients. The term “concept” has proven to be notably challenging to define, with variations in meaning according to different authors’ perspectives on the subject. In this paper, we offer a definition of “concept” as an abstract or general idea that serves as a mental construct or phenomenon.

Here, our primary objective is to examine the phenomenon of self-deception using Rodgers’ evolutionary method (Rodgers, 1989) of concept analysis as our guiding framework. To achieve this, we employ a structured process that unveils the intricacies of self-deception within the context of nursing clinical practice. Our process commences by establishing a comprehensive backdrop that highlights the significance of our chosen topic. We also provide an overview of related concepts intertwined with self-deception. This contextual foundation enables us to navigate the subsequent analysis with a clear understanding of the fundamental distinctions. Moving forward, we explore the antecedents, attributes, consequences, and real-world instances of self-deception, drawing insights from a review of relevant literature. These components collectively contribute to a holistic comprehension of self-deception and its potential implications for clinical nursing practices. Lastly, our discussion and conclusion segments synthesize the findings and extrapolate the implications of self-deception within the context of clinical nursing. By adhering to this methodological approach, we aspire to illuminate the intricate facets of self-deception, equipping readers with a comprehensive understanding of its significance and far-reaching effects in the domain of nursing clinical practice.

## Context

Power dynamics in healthcare settings have been critical in nurses’ experiences during the COVID-19 pandemic. This may have led to tensions between their professional knowledge, ethical decision-making, and sense of voice, resulting in moral distress (MD). MD is a psychological and emotional response that occurs when a person, in this case, a nurse, knows the morally right action to take in a situation but is unable to do so due to institutional or hierarchical constraints, which may contribute to nursing shortage due to burnout and attrition (Oh & Gastmans, 2015). MD is a well-known phenomenon among nurses, and the COVID-19 pandemic increased the risk of experiencing it (Schlak et al., 2022). Indeed, studies have shown that nurses are at a higher risk of psychological distress, including depression, anxiety, and burnout, during pandemics (Gee et al., 2022; Oh & Gastmans, 2015). Nurses who perceive their work environment (and practices) as morally distressing, such as performing invasive or unnecessary treatments, have significantly higher odds of considering leaving their positions (Sheppard et al., 2021). Past research has also examined how nurses may draw upon moral resilience, which refers to an individual’s ability to maintain or restore their moral integrity in the face of moral ambiguity, uncertainty, distress, or adversity (Young & Rushton, 2017), to overcome these challenges. Morley et al. (2022) found that some nurses faced various ethical challenges regarding patients dying alone, surrogate decision-making, imbalance (of power), and injustice between professionals and were able to provide “good” care by drawing upon their strength and ethical values. However, in exploring nurses’ experiences of speaking up about concerns during the pandemic, Abrams et al. (2023) highlighted that nurses felt they had no agency or choice. Speaking up was often met with disciplinary actions or reprimands. Nurses were “at risk” if they raised issues, and trust was largely absent during the pandemic; speaking up was met with little change or support. Elisha et al.’s (2022) and Shir-Raz et al.’s (2022) findings revealed that health professionals critical of the pandemic management and treatments reported feeling repressed and censored through online content control, defamatory statements, retractions, denial of research grants, dismissal calls, and hearings. As a result, many refrained from expressing critical positions; a *chilling effect*, understood as the suppression or discouragement of a certain behavior, usually the exercise of free speech, due to fear of potential legal, social, or political repercussions, on their willingness to speak out and advocate for their patients and themselves (Delborne, 2016). Given the historical and taken-for-granted ideas about nurses that emphasize specific traits such as caring and sacrifice (Boulton et al. (2021), by what mechanism do nurses rationalize their behavior and the institutions they work for to protect their ego and avoid uncomfortable truths? More importantly, what is the role of self-deception in nursing practice?

Settling upon a definition of self-deception is a contentious issue. Khalil (2017) notes that “there is no systematic study in the literature of how self-deception differs from other kinds of self-distortion” (p. 539). They found it beneficial to define necessary conceptual distinctions between self-deception with other kinds of self-distortion that the literature often confuses. Although “deception” can lead to self-deception, self-deception “is an act whose audience, in the final analysis, is the conscious self. Spectators are not essential” (p. 544). Deception appears in nursing literature as a gesture to spare a patient or family unnecessary grief (de Vries & Timmins, 2016a). The concept of “delusion” is often used and can be defined as “deceiving oneself about a clear-cut fact, while delusion is deluding oneself about an aspiration or a goal. The aspiration or goal is never, by definition, a clear-cut fact” (p. 542). A literary search of the concept of delusion in the nursing literature yields references to mental illnesses such as dementia patients. The concept of “moral licensing” which is about one’s “moral standing or moral capital” as opposed to self-deception, which is “concerned with facts, and hence should not be conflated with moral licensing” (p. 542), also appears to be foreign to nursing literature. “Cognitive dissonance reduction,” where “cognitive dissonance reduction is understood as ‘bridging’ the painful gap between material utility and ethical utility” (p. 542), is the expression most closely related to self-deception. However, “cognitive dissonance is not necessarily about ex-post justification, while self-deception is” (p. 555). Cognitive dissonance appears in nursing literature in various contexts, including coerced psychiatric measures.

We contend that understanding self-deception in nursing is highly relevant to advanced nursing practice due to its implications for ethical decision-making, moral resilience, and the well-being of nurses. In the context of the COVID-19 pandemic and the display of power dynamics within healthcare settings, understanding self-deception becomes even more critical. Given that nurses may rationalize their behavior or refrain from speaking out due to fear of retribution, institutional power dynamics, or social expectations, understanding self-deception can help nurses identify these tendencies, challenge them, and develop strategies to effectively voice their concerns and advocate for positive change. Understanding self-deception not only contributes to the advocacy of the nursing profession but also fosters self-awareness, critical reflection, and the ability to examine one’s own biases and assumptions. This promotes personal and professional growth, enabling nurses to provide high-quality care, engage in evidence-based practice, and contribute to the advancement of the nursing profession.

## Rodgers’ Evolutionary Method

### Overview of the Method

Rodgers (1989) identifies the purpose of concept analyses in bringing clarity to concepts, in this case, *self-deception* in nursing practice. This analysis is performed with dispositional theory in mind, where concepts are presented as “habits or capacities for certain behaviours” (p. 11). The result is expected not to have rigid and distinct boundaries but will instead reflect “resemblances or commonalities in the use to the word or concept” (p. 21) of self-deception. The selection of an approach for concept development reflects the internal conceptual problem, which Laudan (Rodgers, 1989) classifies as “problems related to the lack of clarity of basic concepts” (p. 28).

Rodgers’ method is coined “evolutionary” because the concepts are in constant dynamic development shaped by the context in which they are used over time (Tofthagen & Fagerstrøm, 2010). A concept’s context is understood differently depending on discipline, culture, and the period of time. Given that this analysis incorporates sources from diverse fields outside of nursing, Rodgers’ method is a fitting and appropriate approach to foster interdisciplinary exploration and enhance understanding of concepts. The method’s decisive advantage is its clear and systematic process of describing and explaining concepts applicable to the nursing field (Tofthagen & Fagerstrøm, 2010). Although this paper aims to define self-deception in nursing practice, “analysis merely indicates a direction for further research and does not provide a definite conclusion” (Tofthagen & Fagerstrøm, 2010, p. 22). Rodgers’ method includes six steps: (a) identification of the concept and associated expressions; (b) selection of domain for data collection; (c) data collection guided by research questions; (d) data analysis; (e) identification of an exemplary case, and (f) discussion for further development of the concept.

### The Context of the Concept

According to Rodgers (1989), the choice of a concept should serve a purpose in advancing or addressing a cause, in this case, the nursing clinical practice. It also clarifies a conceptual definition in instances where similar concepts are a source of confusion. Different authors may express the same concept differently; therefore, it is essential to consider the various terminologies that may have been used to describe the same concept (Tofthagen & Fagerstrøm, 2010). Self-deception falls under the umbrella of self-distortion. Other distortions include deceptions, delusions, manipulation, moral licensing, cognitive dissonance, and introspection illusion (Khalil, 2017). As Rodgers’ states, the evolutionary method of analysis “is primarily a means of identification, not imposing any strict criteria, expectations, or view of reality on the concept, but simply seeing what is common in the existing use of the concept” (Rodgers, 1989, p. 333).

### Collection of Material for Concept Analysis

Inclusion criteria were scholarly papers, book chapters, and English language publications between 2000 and 2022. Exclusion criteria included dissertations, editorials, or response pieces. An interdisciplinary approach was used to include studies from different fields including nursing, health, psychology, and philosophy, as foundational material for understanding the mechanisms of self-deception. A total of four databases were used: CINHAL, Medline, PubMed, and PsycINFO using the search subject search terms “self-deception” AND the truncation “nurs*” to capture “nurse,” “nurses,” and “nursing.” Once the search was completed, duplicates were removed, title and abstracts screened, and then full texts were reviewed against inclusion and exclusion criteria.

### Analysis

Rodgers’ evolutionary method (Tofthagen & Fagerstrøm, 2010) involves analyzing concepts by focusing on surrogate terms, antecedents, attributes, consequences, and examples. In this analysis, surrogate terms were excluded as none accurately describe self-deception in nursing clinical practice. The identified themes were organized and compared for pattern emergence.

## Results

**Figure 1.**
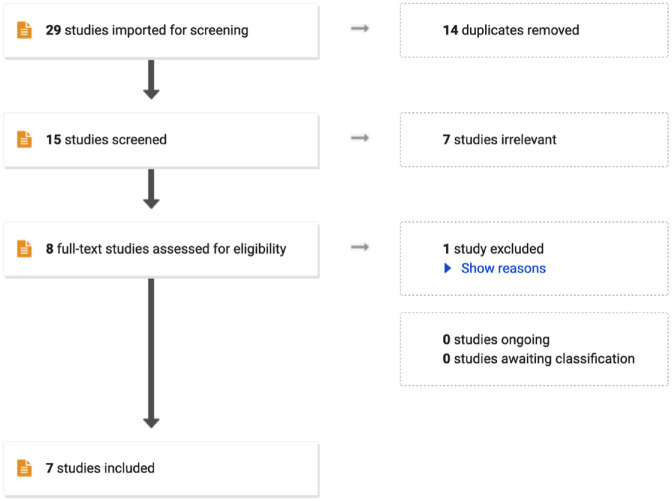
Article search and exclusion process.

In all, 29 references were imported into *Covidence* for screening and 14 duplicates were removed. In all, 13 papers were screened against title and abstract, and 7 papers were excluded, including editorials, response letters, or the subject being patients’ or student experience rather than the practicing nurses. Six papers were assessed for full-text eligibility where one study was excluded for being about communication strategies as opposed to nursing experience. In total, seven sources were retained for this concept analysis (see Figure 1).

Five papers relating to self-deception in nursing practice were identified, and two papers from the field of psychology were included to provide valuable insights into the mechanisms and implications of self-deception (see Figure 1). The first paper from the field of nursing by de Vries and Timmins (2016a) explores the interconnectedness of deception and self-deception in healthcare, emphasizing their role in protecting patients and reducing cognitive dissonance. David (2000) examines nursing’s gender politics, identifying self-deception as a central concept that perpetuates professional mediocrity and limits nurses’ freedom. Paley (2002) discusses caring as a “slave morality,” drawing on Nietzschean themes to analyze the drawbacks of caring in terms of favoritism and injustice. Garrett’s (2016) essay critiques postmodern metaphysical antirealist ideologies in nursing theory as a form of self-deception, negatively impacting the nursing profession. Lastly, Roberts (2016) addresses poor patient care, attributing it to self-deception, particularly Sartre’s notion of “bad faith.” The first paper from the field of psychology by Tenbrunsel and Messick (2004) explores how self-deception facilitates unethical behavior by allowing individuals to behave self-interestedly while falsely believing they upheld moral principles, while the second paper, by von Hippel and Trivers (2011), discusses the evolution and psychology of self-deception, highlighting its role in interpersonal deception and the benefits of self-deceptive self-enhancement.

### Antecedents

The context and circumstances in which the concept of self-deception emerges reveal four key antecedents: (a) contradiction/cognitive dissonance, (b) stress/anxiety, (c) power imbalance, and (d) deceit. Each will be defined below.

#### Contradiction/Cognitive Dissonance

Cognitive dissonance refers to the discomfort individuals feel when they try to reconcile conflicting demands or engage in activities that go against their beliefs. This creates a perception of incompatibility or dissonance (Festinger, 1957). The avoidance of the “pain” caused by dissonance is at the root of the dissonance reduction and opportunity of self-deception, giving priority to welcome over unwelcome information; “typically, when juggling contradictory statements and evidence, self-deception may creep in before deception of others. We like to hold on to narratives that reduce cognitive dissonance” (de Vries & Timmins, 2016a, p. 168).

#### Stress/Anxiety

Unattainable professional demands because of increasingly complex healthcare environments give rise to heightened stress. This stress burdens healthcare workers to adapt to their work environments and deliver care by “cutting corners,” often at the expense of patient-centered care (de Vries & Timmins, 2016a). Self-deception is an effort to resist the unwelcome truths of such practice (Garrett, 2016). Anxiety is also generated from a culture of silence that fears the repercussions of engaging in critical thought and choice-making. The professional obligation to raise poor patient care concerns competes with the perceived organizational unresponsiveness to reporting such concerns (Roberts, 2016).

#### Power Imbalance

David (2000) explains that nursing, being a predominantly female-gendered profession, grapples with power imbalances inherent in a gender-class-conscious society. Nurses desire to believe they are in charge but are bound to the authority and power dynamics dictated by patriarchal norms. The perception of nursing as a female profession perpetuates societal beliefs and facilitates social control over nurses. Paley describes the philosophy of “caring” as a product of subservience, leading to an inverted sense of self (Paley, 2002).

#### Deceit

Self-deception may be proceeded by a desire to deceive others. By convincing themselves that are not dishonest, nurses can better deceive others without being detected and eliminate the discomfort that comes with knowing deceit (de Vries & Timmins, 2016a). The deceit of self is also possible by preventing unwanted information from being encoded in the first place. For example, a nurse will stop gathering information on a favorable situation they find themselves in; however, they will seek more information if the situation is not in their favor (von Hippel & Trivers, 2011). “By avoiding or disguising the moral implications of a decision, individuals can behave in a self-interested manner and still hold the conviction that they are ethical persons” (Tenbrunsel & Messick, 2004, p. 225).

### Attributes

The characteristics of self-deception in nursing practice can be understood by examining its attributes. Self-deception in this context is characterized by cognitive distortions, which are identifiable errors in thinking that result from processing information in adaptive or maladaptive ways (Yurica & DiTomasso, 2005). The six attributes of self-deception are as follows: (a) discomfort/dissonance reduction, (b) deception of others/disguising our ethical violations, (c) distorted reality, (d) biased information, (e) ethical fading, and (f) self-serving. Each will also be defined below.

#### Discomfort/Dissonance Reduction

Self-deception serves as a mechanism to alleviate discomfort triggered by conflicting demands or cognitive dissonance. It allows nurses to adapt their perspective and no longer consider their actions as deceptive, reducing feelings of guilt, shame, or regret (de Vries & Timmins, 2016a). Nietzsche, as described by Paley (2002), suggests that resentment toward the dominant medical paradigm can lead to a “slave revolt” and the emergence of exalted counter values. The medical establishment’s perceived positive qualities such as positivism, reductionism, and mechanism are stigmatized as wicked, inhuman, and manipulative. This “imaginary revenge” derives power from a self-deceptive belief that the weakness inherent in our status as nurses is virtuous and good (Paley, 2002).

#### Deception of Others/Disguise Our Ethical Violations

The classic form of self-deception is convincing oneself that a lie is true (von Hippel & Trivers, 2011). We tend to adopt false memories as a means of self-deception, enabling us to maintain a sense of inner peace when we lie or hold unethical beliefs. Deception and self-deception often go hand in hand, as self-deception enhances the effectiveness of deception itself (de Vries & Timmins, 2016a). What may start as deception of others can evolve into self-deception to avoid detection, possibly serving as an evolutionary adaptation. Believing the lie helps nurses avoid the discomfort of honesty and prevents the display of telltale signs of deceit (de Vries & Timmins, 2016a; von Hippel & Trivers, 2011). Self-deception also allows nurses to deceive others into perceiving them as better than they truly are, whether it be in terms of ethics, qualifications, or strength. Moreover, even when self and others accurately recall one’s past wrongdoings, it is still possible to avoid facing the whole truth by reconstructing or rationalizing the motives behind one’s actions to make them more socially acceptable (von Hippel & Trivers, 2011). Self-deception influences decision-making processes that eliminate negative ethical characterizations or distort them into positive ones (Tenbrunsel & Messick, 2004).

#### Distorted Reality

According to David (2000), nurses engage in creating illusions to distort their experiences and deny the reality of their secondary gender-class existence. These illusions serve as a means for them to embrace their peripheral positions within society. Distorting reality allows these beliefs to persist, even in the face of contradictory evidence. Garrett (2016) argues that this distortion of reality lies at the core of nursing’s acceptance of mystical or critical theories, which come at the expense of embracing modern reductionist science. He contends that the belief that such ideas contribute to the advancement of the nursing profession lacks evidence and is instead rooted in delusion and self-deception. Nietzsche viewed the “caring paradigm” as a distortion that reverses reality, representing an anti-scientific path that rejects the medical model as evil and transforms weakness into a basis for self-congratulation (Paley, 2002).

#### Biased Information

Whether consciously or unconsciously, individuals tend to actively choose information that reduces cognitive dissonance, reinforces their existing beliefs, and evokes positive emotions (de Vries & Timmins, 2016a). Biased information search strategies and interpretive processes are employed as means to prioritize welcoming information over unwelcome information, aligning with personal goals and motivations (von Hippel & Trivers, 2011, p. 2). In the pursuit of reducing dissonance, nurses may refrain from conducting further information searches, as they might come across information that contradicts their goals or the objectives of their institution. Selective search strategies are employed to minimize exposure to unwelcome information, while selective attention focuses on preferred information and avoids challenging perspectives (von Hippel & Trivers, 2011).

#### Ethical Fading

Ethical fading is a gradual process that transforms morally wrong behavior into socially acceptable actions by diminishing the moral significance of the situation. Through re-labeling and concealing the ethical implications, individuals can reduce their sense of moral responsibility (de Vries & Timmins, 2016a; Tenbrunsel & Messick, 2004). This process involves psychological numbing, often referred to as the slippery slope, where repeated exposure to ethical dilemmas weakens self-reproach and diminishes the ethical considerations associated with such situations (Tenbrunsel & Messick, 2004). Ethical fading can lead to unreflective and potentially more unethical behavior as each small step away from ethical practices, if perceived as not qualitatively different, contributes to a progression toward unethical activities (Tenbrunsel & Messick, 2004, p. 228). Another technique employed in ethical fading is the use of euphemisms. By substituting morally repugnant language with abstract and neutral terms, individuals can distort the narratives surrounding ethical dilemmas or distressing situations (de Vries & Timmins, 2016a; Tenbrunsel & Messick, 2004). This technique is reminiscent of the Orwellian Newspeak (Orwell, 1984).

#### Self-Serving

Self-deception is recognized by several nurse authors as a strategic tool used for self-serving purposes. By embracing self-deceptive beliefs, individuals can more easily engage in activities they enjoy or gain social and economic advantages (Garrett, 2016). Using a Nietzschean approach, one could argue that the concept of “caring” in nursing represents an unconscious desire for power over others, allowing nurses to compensate for the oppression they face from the medical profession and elevate their self-esteem (Paley, 2002). Self-deception conceals the self-serving nature of one’s actions, enabling individuals to act in their own self-interest while falsely believing they are upholding moral principles (Tenbrunsel & Messick, 2004). By convincing oneself and others of falsehoods, self-deception becomes a mechanism for personal gain and social recognition (von Hippel & Trivers, 2011).

### Consequences

Three consequences of self-deception were identified: (a) infighting, (b) perpetuation of the (subordinate) status quo, and (c) unaccountability and the decline of care.

#### Infighting

David (2000) observes that self-deception undermines nurses and increases their susceptibility to horizontal violence, manifested as aggressive behaviors aimed at hindering coworkers, advancing personal agendas, and alleviating tensions resulting from situational subservience. Garrett (2016) argues that nursing’s alignment with the social sciences, instead of the physical sciences, fosters polarized debates concerning the theory-practice gap and promotes anti-evidence-based-practice discourse.

##### Perpetuation the (Subordinate) Status Quo

Adopting multiple and relativistic ways of knowing to support practice exacerbates professional fragmentation (Garrett, 2016). The failure to acknowledge self-deception restricts nurses’ autonomy and perpetuates their subordinate status within the profession. Nurses may deceive themselves into believing that they desire the circumstances imposed upon them. This self-deceit reinforces gendered behaviors and contributes to the gender politics prevalent in professional and service delivery organizations (David, 2000). By actively participating in the medical hegemony and adhering to traditional physician-nurse roles, nurses demonstrate their subservience. This perpetuates a cycle where the belief that increasing educational requirements for entry into the profession is not a priority or open to further debate becomes ingrained. Insufficient education sustains subordination, trapping nurses in non-theoretical, lower-class practices. Consequently, the public’s understanding of the nursing role remains inadequate, and the dominant interests of the patriarchy persist. Many nurses prepared in diploma and associate degree programs are enabled by self-deception to disregard the systemic support that keeps them in lower-level work practices (David, 2000). Moreover, adopting determinism, the belief that our behavior is determined by situational factors beyond our control, undermines our freedom and capacity for critical reflection and decision-making in patient care and nursing practice (Roberts, 2016). Von Hippel and Trivers (2011) support the notion that those in subordinate positions are prone to self-deception, as they may have various motivational reasons to support the status quo, even when they are clear losers in the existing system with little chance of improving their situation.

#### Unaccountability and the Decline of Care

Self-deception undermines nurses’ ability to make ethical decisions, as it allows them to distance themselves from the ethical demands of a situation, diminishing their significance (Tenbrunsel & Messick, 2004, p. 231). In addition, when they assume that a system is error-proof, they tend to overlook the environmental factors that contribute to unethical behavior, thereby reducing the likelihood of improving the system (Tenbrunsel & Messick, 2004). The ability of nurses to recognize and report instances of inadequate care is crucial for initiating meaningful changes, not only at an individual level but also at a structural level. Without this ability, blame is often shifted between individuals and organizations, hindering necessary improvements (de Vries & Timmins, 2016a). Furthermore, the complexity of modern healthcare systems and the dispersion of responsibilities make it challenging for nurses to fully comprehend the cumulative impact of gradual declines in the quality of practice. Unfortunately, these declines often go unnoticed until the point when damage to patient care becomes evident (de Vries & Timmins, 2016a).

### Exemplar

In a more practical context, de Vries and Timmins (2016a) shed light on the impact of self-deception on the provision of care. They describe a situation where nurses face a dilemma between delivering personal and compassionate care and meeting the constraints and demands of their tasks. This creates a discomforting scenario where self-deception comes into play. According to de Vries and Timmins (2016a), “self-deception” allows understaffed and overworked nurses to deliver minimal care as a substitute for the real thing. Convincing ourselves that 30 s of listening to a patient “ticks the box” begs the question, “are we collectively deceiving ourselves by setting person-centeredness as the standard for good care?” (p. 167). This example illustrates the conditions of contradiction, stress, and deceit that contribute to self-deceptive distortions, such as disguising ethical violations and ethical fading. The consequences of this self-deception include perpetuating the decline of care, fostering unaccountability, and contributing to the erosion of care, a concern also addressed by Roberts (2016).

### New Definition

Our analysis reveals a conceptual definition of self-deception as a means by which nurses cope with or justify nurses their subordinate position within the hierarchical structure of healthcare. Nurses may distort their own beliefs, needs, or values to align with the dominant power structures or to conform to the expectations placed upon them. They may convince themselves that their subservience and conformity are necessary for the smooth functioning of the healthcare system, or they may downplay their own expertise and contributions.

## Discussion

This concept analysis of self-deception in nursing practice aims to improve understanding and mitigate its negative consequences. Focusing on nurses’ experiences in challenging situations, the analysis explores how self-deception is often employed as a coping strategy. The context is set within the power dynamics of healthcare, using the COVID-19 pandemic as a prime example, which might have increased MD and psychological distress among nurses. Nurses may face contradictory demands or actions that go against their beliefs, leading to cognitive dissonance. Self-deception can arise as a defense mechanism to reduce this dissonance, prioritize information that aligns with their preferred narrative, and embrace distorted beliefs to deny their peripheral position in society.

The selected literature draws heavily from philosophy and theories, such as Sartre’s notion of “freedom” (Roberts, 2016) and Nietzsche’s concept of “slave morality” (Paley, 2002). Furthermore, feminist theory (David, 2000) and the post-positivist evidence-based paradigm (Garrett, 2016) are presented in contrasting positions. Notably, self-deception operates within power dynamics, both as a tool of oppression and as a means of self-oppression. Scholars have observed power relations in nursing as conflicting, where control is exerted and experienced (Gastaldo & Holmes, 1999). According to Foucault, power is fluid, circulating among individuals and shaping their actions toward others, highlighting its relational nature (Holmes & Gastaldo, 2002). Von Hippel & Trivers (2011) caution that system-justifying beliefs, a form of self-deception, may be imposed by those in higher social positions to legitimize the lower status of individuals, inhibiting social change. This suggests that self-deception’s consequences extend from intrapersonal to societal levels (Von Hippel & Trivers, 2011). The broad set of circumstances in which self-deception has been written about in such a short number of texts speaks to the ubiquitous implications of self-deception in nursing. It suggests a need to investigate the concept further. Deweese-Boyd (2016) succinctly summarizes self-deception as

a problem of existential concern, since it suggests that there is a distinct possibility that we live with distorted views of ourselves, others and the world that may make us strangers to ourselves and blind to the nature of our significant moral engagements. (para. 2)

Nursing is arguably built on its sense of moral engagement; hence, making it a critical philosophical exploration to be had. Recognizing the conditions that give rise to discomfort, distress, and cognitive dissonance can catalyze interventions aimed at mitigating or preventing self-deception and its resulting consequences. Moreover, gaining a comprehensive understanding of the mechanisms that sustain self-deception provides valuable insights for addressing and advocating for nurse well-being and ethical practice.

Considering the current crisis such as nursing shortage and burnout experienced in nursing, the findings of this concept analysis highlight the importance of further exploration and discussion of self-deception in nursing clinical practice. Subsequent studies in the field would greatly benefit from delving deeper into the implications of self-deception, particularly in the context of contemporary challenges faced by nurses. This future research would contribute to addressing the unique needs and promoting the well-being of nurses in these demanding times. An equally pertinent inquiry revolves around the actions of those in leadership roles, such as Deans, managers, and Chief Nursing Officers. Do nurses in positions of power employ self-deception to rationalize their treatment of other nurses, potentially perpetuating behaviors associated with “oppressed group” dynamics or “nurses eat their young” culture? Furthermore, can self-confidence in one’s leadership ever cross into the realm of self-deception? In addition, examining the role of pseudo-psychological coping strategies like “reframing” becomes crucial when they are used to manipulate or create altered perceptions of past events. In raising these questions, we delve into the intricate interplay of power dynamics, coping mechanisms, and ethical considerations within the nursing profession, shedding light on the complexities of self-deception at various levels of the healthcare hierarchy.

The limited number of papers about self-deception in the field of nursing is the main limitation of this conceptual analysis. In addition, research that addresses self-distortions in nursing is predominantly associated with patient experience (particularly dementia, end of life, and grief) as opposed to nurses’ lived experience. Therefore, support text from psychology was limited to the two hits derived from PsycINFO to keep the information concise and nursing focused.

## Conclusion

The phenomenon of self-deception in nursing clinical practice highlights profound tensions between different paradigms and expectations within the healthcare system. These tensions stem from the power dynamics and subservience that nurses often face, which can hinder their ability to advocate for themselves, their patients, and the nursing profession. To address this tension, it is crucial to recognize and challenge the prevailing paradigms that perpetuate self-deception and marginalize nurses altogether. A critical examination of power dynamics within healthcare and the promotion of a more equitable and collaborative approach to healthcare delivery is imperative. Nurses need to develop a heightened awareness of the potential for self-deception, challenge societal and institutional expectations, and actively assert their professional autonomy. By recognizing their own value, advocating for their rights, and seeking opportunities for empowerment, nurses can contribute to dismantling systems of subservience and promoting a more balanced and patient-centered healthcare environment.
